# Do Implicit Motives Influence Perceived Chronic Stress and Vital Exhaustion?

**DOI:** 10.3389/fpsyg.2018.01149

**Published:** 2018-07-04

**Authors:** Jessica Schoch, Emilou Noser, Ulrike Ehlert

**Affiliations:** ^1^Clinical Psychology and Psychotherapy, University of Zurich, Zurich, Switzerland; ^2^University Research Priority Program—Dynamics of Healthy Aging, University of Zurich, Zurich, Switzerland

**Keywords:** implicit motives, affiliation, achievement, power, chronic stress, vital exhaustion, men's health

## Abstract

Vital exhaustion (VE) results from the experience of chronic stress. However, research on stress types and their relation to VE is rare. Moreover, the role of implicit motives in these processes has not yet been investigated. Analysis included 101 vitally exhausted men aged 40–65 years. Participants provided self-report data on their experience of chronic stress and social support. Subtypes of work-related and social stress were positively associated with VE. Implicit affiliation and achievement motives were linked to social support and chronic stress, and indirectly to VE. Moreover, they moderated the relationship between stress and exhaustion. In conclusion, implicit motives are key factors in the stress process: They are involved in an individual's experience of stress and stress-related consequences for mental health.

## Introduction

Vital exhaustion (VE) is characterized by a lack of energy, feelings of demoralization, and irritability (Appels and Mulder, 1988). Over the last decade, it has received considerable research attention due to its adverse effects on health and well-being (Prescott et al., 2003; Appels, 2004; Tselebis et al., 2011; Rafael et al., 2014). In particular, studies have found VE to be an independent risk factor for cardiovascular disease (CVD; Appels and Mulder, 1989; Frestad and Prescott, 2017). Moreover, men are at greater risk to suffer from CVD (Jousilahti et al., 1999).

The cause of VE is still not fully understood. It is hypothesized that it develops as a result of failed adaptation to chronic stress (van Diest and Appels, 2002; Noser et al., 2018). When investigating the stress-strain relationship, it is critical to assess the specific type of stress. However, to date, only two studies have examined the association between chronic stress and VE. A study by Schnorpfeil et al. (2002) found that VE was linked to perceived work stress, including workload and qualitative demands at work. Another study found that VE was further positively related to chronic social conflicts at work (Falger and Schouten, 1992).

Besides the type of stress, social support should also be taken into account, since the perception of high social support has numerous benefits for physical and mental health (Uchino, 2009; Taylor, 2011). Conversely, low social support is considered to be stressful and detrimental for an individual's well-being. In one study, men with severe VE reported the lowest levels of social support (Noser et al., 2018). Likewise, low perceived support from others was found to contribute to VE and heart failure (Cené et al., 2012). In summary, work-related and social stress, as well as low social support, are related to negative health states, including VE.

With respect to the psychobiological stress response, there is large interindividual variability, which is partly attributable to individual differences in personality (Bolger and Schilling, 1991; Parkes, 1994; Sapolsky, 1994; Spector and Bruke-Lee, 2008). Personality characteristics influence the probability that an individual will seek out certain situations and be exposed to potential stressors (Bolger and Zuckerman, 1995; Bresin and Robinson, 2015). They further affect how an individual appraises stressors. Therefore, individuals with specific personality characteristics might be more or less prone to experience stress in the first place. Similar assumptions hold true for the influence of personality on social support (Pierce et al., 1997; Swickert, 2009). Moreover, as the direct associations between the experience of stress and subsequent health states are only small to moderate, personality may function as an intervening variable (Code and Langan-Fox, 2001). Hence, individual differences in personality may render a person more vulnerable or resilient to the consequences of stress (Cohen and Edwards, 1989; Parkes, 1994; Lee and Ashforth, 1996; Wiebe and Smith, 1997).

In conclusion, personality characteristics might have a direct impact on the experience of stress and social support. Furthermore, they are potential moderators of the stress-strain relationship. These assumptions have already been confirmed for traditional personality traits such as the Big Five (e.g., Parkes, 1990; Lakey and Dickinson, 1994; Mroczek and Almeida, 2004; Ebstrup et al., 2011).

Previous research has largely neglected motivational aspects of personality, namely implicit motives, which represent the need to experience positive affect in response to specific incentives (Schultheiss, 2008). Conversely, individuals will respond with negative affect to motivational disincentives. Studies have concentrated on the assessment of three social motives: affiliation, achievement, and power (Schultheiss, 2008). Affiliation motive is defined as a concern with warm, friendly relationships, while individuals motivated for achievement strive to attain a standard of excellence (McClelland et al., 1953). The implicit motive for power represents the need to have impact, control, or influence on others (Winter, 1973). Since implicit motives seem to be shaped during the preverbal phase in childhood, they are largely inaccessible to consciousness and are best assessed using projective measures (McClelland, 1989; Thrash et al., 2012).

The strength of each implicit motive influences processes of perception, cognition, affect, and subsequent behavior (McClelland, 1987; Schultheiss and Brunstein, 1999). Arguably, implicit motivation is involved in a person's appraisal and thus, their experience of stress. Depending on the type and strength of each implicit motive, an individual could perceive a situation as a threat to an underlying motive or as an opportunity for motive satisfaction. The former type of appraisal would cause a person to experience stress. To our knowledge, so far, only one study has tested this assumption. In a sample of fathers, we operationalized the experience of stress by men's ratings of their perceived constraint due to fatherhood. The implicit motive for affiliation was related to less perceived stress, whilst implicit power motive had the opposite effect (Ruppen et al., 2016). Surprisingly, a literature search did not yield any studies investigating the role of implicit motives in social support.

Implicit motives might further affect the relationship between the experience of chronic stress and an individual's subsequent health. In contrast to the previous assumption, this hypothesis has already been tested from the early beginnings of motivational research (see McClelland, 1989 for an overview). Implicit motives are aroused and become effective in situations that are relevant for the specific motivational domain (McClelland et al., 1953). For example, the experience of social stress should be threatening for individuals highly motivated for affiliation, as it bears the risk of frustrating this particular motive. Therefore, implicit motives might influence stress-related health consequences depending on the type of stress. Several experimental studies have investigated the moderating role of implicit motives. A high implicit motive for affiliation was found to dampen the effect of a social stressor on the subsequent cortisol response, while similar effects were not found in response to a physical stressor or in a control condition (Wegner et al., 2014). Presumably, individuals high in affiliation motive did not perceive the incentives inherent in the social stress condition (such as social evaluation) as threatening (Wegner et al., 2014), but rather regarded them as opportunities to satisfy their affiliative need. In a similar study, high levels of implicit achievement motive predicted an attenuated cortisol response after a competitive task, independent of whether participants won or lost (Schultheiss et al., 2014). The same results were obtained for a psychosocial stress situation compared to a control condition (Schultheiss et al., 2014). Since individuals high in achievement motive take pleasure in engaging in a task *per se*, due to their preference for mastering challenges, they are less likely to interpret such a situation as threatening or stressful (Lazarus and Folkman, 1984; Reeve et al., 1987). In comparison, participants with high levels of power motivation showed increases in cortisol levels when they had lost a contest, but not when they had won (Wirth et al., 2006). Apparently, the frustration of their need for power resulted in a biological stress response.

Previous studies show that when studying the consequences of the stress experience, one should consider specific types of stress rather than a general measure of chronic stress. Therefore, in a first research question, we will examine different subtypes of work-related and social stress and their relation to VE in a sample of vitally exhausted men.

We further plan to investigate the role of implicit motives in these relationships. Implicit motives might directly or indirectly influence the experience of social support, chronic stress, and VE. The second research question addresses the direct effect of implicit motives. Implicit affiliation motivation is hypothesized to be linked to higher perceived social support, and thus lower chronic stress and VE. The analyses regarding achievement and power motives remain largely exploratory in nature. For the third research question, we will investigate whether implicit motives are moderators of the stress-strain relationship. Implicit motives interact with incentives relevant for the given motive. We assume that the interaction between a motivational domain and a specific incentive, that is a specific subtype of chronic stress, determines the consequences for an individual's health. We hypothesize that implicit affiliation motive leads to greater exhaustion in response to social stress. We further expect a buffering effect of implicit achievement motive on the association between work stress and VE. In contrast, higher implicit power motivation in combination with work stress should be more detrimental for the individual health.

## Materials and methods

### Participants and protocol

Data were collected in the framework of the Men Stress 40+ Study, a large research project investigating the effects of stress in men aged 40–75 years (Noser et al., 2018). Participants were recruited in the German-speaking part of Switzerland between January and September 2016 through flier distribution, newspaper announcements, mailing lists, and online advertisements. Participants had to be male, aged 40–75 years, and fluent in the German language. Exclusion criteria were acute or chronic mental or physical illness, intake of psychotropic medication or illegal drugs in the past 2 months, being in treatment for mental disorder in the last 6 months, currently undertaking hormone replacement therapy, or consumption of more than two alcoholic units per day. Since the project aimed to investigate biopsychosocial risk and protective factors in vitally exhaustion men, participants were screened for signs of VE, and the cut-off score for study inclusion was set at ≥4 on the Maastricht Vital Exhaustion Questionnaire (Kopp et al., 1998) which equals at least mild signs of VE.

The local Ethics Committee of Zurich approved the study protocol before data collection. All participants provided written informed consent. Psychometric data were gathered online. Participants were invited to a laboratory session at the Department of Clinical Psychology and Psychotherapy at the University of Zurich, where they wrote short texts for the assessment of implicit motives. The original sample consisted of 123 men. We excluded thirteen men because they either did not provide a text for each picture cue or their texts did not meet the required word count of at least 30 words. A further nine men were excluded because they could not report work-related stress due to being retired. This resulted in a final sample of 101 participants.

### Measures

#### Perceived chronic stress

Perceived chronic stress was assessed with the short form of the Trier Inventory for the Assessment of Chronic Stress (TICS-2-K; Schulz et al., 2004). Participants were asked how often they had experienced the described stressful situations in the past 3 months. Answers were given on a 5-point Likert scale ranging from 0 (“never”) to 4 (“very often”). An example situation is: “Times when none of my tasks seem meaningful to me” (Schulz et al., 2004). The questionnaire consists of 30 items that fall into nine different subscales assessing chronic stress in the work and social environment: work overload, social overload, excessive demands at work, lack of social recognition, work discontent, social tension, performance pressure, lack of social contact, and chronic worrying. Additionally, a general screening scale for perceived chronic stress composed of six items can be used for an initial assessment.

#### Perceived social support and support seeking

Perceived social support and support seeking were measured with two subscales of the Berlin Social Support Scales (BSSS; Schulz and Schwarzer, 2003). Each subscale comprises eight items, which are rated on a Likert scale from 1 (“strongly disagree”) to 4 (“strongly agree”). Items were averaged to build a score for each subscale, with a range from 1 to 4. An example item for perceived social support is: “There is always someone there for me when I need comforting.” An example item for support seeking is: “Whenever I am worried, I reach out to someone to talk to.”

#### Vital exhaustion

Vital exhaustion was assessed with the short form of the German Maastricht Vital Exhaustion Questionnaire (Kopp et al., 1998). The nine items cover aspects of VE such as fatigue, loss of energy, or irritability. An example item is: “Do you sometimes feel that your body is like a battery that is losing its power?” The symptoms are rated as “no” (score 0), “don't know” (score 1), or “yes” (score 2), resulting in a possible range from 0 to 18.

#### Implicit motives

We used the Picture Story Exercise (PSE; Schultheiss and Pang, 2007) to produce text samples, which were then coded for implicit motive dispositions in the domains of affiliation, achievement, and power. Participants were shown the following six picture cues for 10 s each in a randomized order: boxer (McClelland and Steele, 1972), nightclub scene (McClelland, 1975), ship captain, trapeze artists, women in laboratory, and couple by river (Smith, 1992). They were asked to take 4 min to write an imaginative story about the characters shown in the pictures. We used standard instructions and procedures as described in Smith (1992). The PSE was administered on a computer during the laboratory session.

Stories were coded for motivational imagery according to the Manual for Scoring Motive Imagery in Running Text (Winter, 1994). Affiliation (*n*Aff) is scored whenever a character expresses concern with establishing, maintaining, or restoring friendly relations such as engaging in affiliative activities or showing sadness about a separation. Achievement (*n*Ach) is scored whenever a character is concerned with achieving a standard of excellence. This includes winning against or competing with others, disappointment about failure, or unique accomplishments. Power (*n*Pow) describes a concern with having impact, control, or influence on others. It is coded whenever a character shows strong, forceful actions, tries to impress, provides unsolicited help, is concerned with reputation, or elicits strong emotions in others. The coder for the present study had attained over 85% inter-rater agreement on expert codings of calibration materials provided by Schultheiss (2015) and underwent several hours of scoring practice prior to coding (Ruppen et al., 2016). Scores from different subscales within a given motive domain were summed up. Word count (*M* = 477.69, *SD* = 151.13) correlated significantly with affiliation (*r*_*s*_ = 0.305, *p* = 0.002), achievement (*r*_*s*_ = 0.341, *p* < 0.001), and power (*r*_*s*_ = 0.421, *p* < 0.001). This significant influence of word count on motive scores was removed by regressing implicit motives on word count, and motive residuals were converted to z-scores for further analyses.

### Statistical analyses

Statistical analyses were performed using the IBM Statistical Package for the Social Sciences (SPSS Version 23) and AMOS 23.0 software package. Statistical significance was defined as *p* < 0.05 (two-tailed). For the first research question, the relationship between implicit motives, social support, chronic stress, and VE was investigated using partial Spearman correlation analyses. In studies on vital exhaustion, analyses are commonly controlled for age and socioeconomic status (see meta-analysis by Frestad and Prescott, 2017). In the present data, control variables age, income, and education (dichotomized) were not significantly correlated with measures of chronic stress, VE, social support, or implicit motives (*p* > 0.05). For reasons of future comparison between studies, these control variables were still included in the analyses.

In the second research question, the direct effect of implicit motives on chronic stress, social support, and VE was investigated with a structural equation model. We applied a maximum-likelihood technique, bootstrapping was set to *k* = 10,000 and 95% bias-corrected bootstrap confidence intervals were computed (Preacher and Hayes, 2008). A model was considered to have a good fit if all path coefficients were significant at the level of *p* < 0.05, χ^2^*/df* was < 2.5 (Bollen, 1989), RMSEA ≤ 0.05 (Steiger, 1990), and CFI > 0.93 (Byrne, 1961). Indirect effects through more than one mediator were analyzed using PROCESS model 6 (Hayes, 2016).

For the third research question, we ran moderation analyses using PROCESS model 1 (Hayes, 2016) to test for the moderating effect of implicit motives on the association between chronic stress subtypes and VE. We controlled for the same sociodemographic variables as used before. All variables were z-standardized prior to entry into the model. As suggested by Aiken and West (1991), the interaction was analyzed by calculating simple slopes between chronic stress and VE at three levels of the moderators: low (one standard deviation below the mean), average (at the mean), and high (one standard deviation above the mean).

## Results

### Descriptive statistics and inter-correlations

Characteristics of the participants are shown in Table 1. Descriptive statistics and inter-correlations among the main study variables for the first research question are reported in Table 2. Implicit motives were not related to VE (*p* > 0.05). VE showed several significant and positive correlations with subscales of chronic stress.

**Table 1:** Characteristics of the sample (*N* = 101); data are presented as mean and standard deviation (for age and annual income) or absolute and relative frequencies.

	**Descriptive**
Age (years)	50.52 (6.58)
Educational attainment	
Vocational training	24 (23.8%)
High school degree	20 (19.8%)
College/university degree	47 (46.5%)
Other	10 (9.9%)
**Employment status**	
Up to 30% employed	1 (1.0%)
31–60% employed	1 (1.0%)
61–80% employed	7 (6.9%)
81–100% employed	92 (91.1%)
**Type of work**	
Perform assigned tasks by myself	3 (3.0%
Working on assigned tasks by myself/with colleagues	41 (40.6%)
Leading a small group of employees/colleagues	42 (41.6%)
Leading a large group of employees/colleagues	15 (14.9%)
Annual income (Swiss Francs)	142,911 (89,978)
**Relationship status**	
Not in a relationship	9 (9.0%)
In a relationship	92 (91%)
**Children**	
No	20 (19.8%)
Yes	81 (80.2%)

**Table 2:** Inter-correlations among implicit motives, support seeking, perceived social support, subscales of chronic stress, and vital exhaustion.

	**M**	***SD***	**1**	**2**	**3**	**4**	**5**	**6**	**7**	**8**	**9**	**10**	**11**	**12**	**13**	**14**	**15**
1. Affiliation	6.77	3.03	1														
2. Achievement	4.31	2.92	0.249*	1													
3. Power	3.59	2.53	−0.005	0.269**	1												
4. Perceived social support	3.36	0.56	0.216*	0.259**	0.038												
5. Support seeking	2.55	0.55	0.235*	0.095	0.084	0.370***											
6. Work overload	7.16	2.33	0.038	−0.200*	0.000	−0.252**	−0.104	1									
7. Social overload	7.45	2.99	0.131	0.028	0.059	−0.200*	0.054	0.356***	1								
8. Pressure to perform	10.60	3.69	0.285**	0.094	0.111	−0.110	0.088	0.244*	0.402***	1							
9. Work discontent	4.22	2.43	−0.218*	−0.019	−0.146	−0.144	−0.262**	−0.018	0.092	0.117	1						
10. Excessive demands at work	3.65	1.99	0.192	−0.158	−0.059	−0.314**	−0.045	0.373***	0.452***	0.253*	0.135	1					
11. Lack of social recognition	4.94	2.42	0.022	−0.116	0.074	−0.232*	−0.315**	0.342**	0.260**	0.183	0.468***	0.336**	1				
12. Social tensions	3.85	1.92	−0.081	−0.155	0.023	−0.306**	−0.051	0.284**	0.275**	0.137	0.250*	0.329**	0.346***	1			
13. Lack of social contact	4.46	2.11	−0.044	−0.274**	−0.108	−0.355***	−0.178	0.377***	0.183	0.205*	0.343**	0.359***	0.343**	0.245*	1		
14. Chronic worrying	5.38	2.35	0.177	−0.133	0.011	−0.383***	0.010	0.475***	0.364***	0.383***	0.159	0.525***	0.349***	0.407***	0.363***	1	
15. Vital exhaustion	11.29	3.59	0.154	−0.140	−0.073	−0.172	−0.142	0.277**	0.041	0.158	0.198	0.230*	0.389***	0.178	0.216*	0.371***	1

*N = 101. Partial spearman correlations. Control variables: age, income, education (dichotomized). Implicit motives are listed in rows 1–3, measures of support seeking and perceived social support are reported in rows 4 and 5, subscales of perceived chronic stress are shown in rows 6–14, vital exhaustion in the last row*.

Range of 1–4 is possible for perceived social support and support seeking; range of 0–12 for the chronic stress subscales work overload, work discontent, excessive demands at work, lack of social recognition, social tensions, lack of social contact, and chronic worrying; range of 0–16 for social overload, range of 0 – 20 for pressure to perform, range of 0–18 for vital exhaustion. SD, standard deviation. Significance levels (two-tailed):

† p < 0.10,

* p < 0.05,

** p < 0.01,

*** *p < 0.001*.

### Structural equation modeling

In order to test the second research question that investigates the relationships between implicit motives, perceived chronic stress, perceived social support, and VE in an overall model, we applied structural equation modeling. We tested a hypothesized structural equation model that included direct paths from each implicit motive to chronic stress, social support, and VE. Additionally, we modeled indirect paths from implicit motives to chronic stress through social support and to VE through social support and chronic stress. The first model with all hypothesized paths fitted the data well, χ^2^(1, N = 101) = 0.109, *p* = 0.741, χ^2^/*df* = 0.109, RMSEA = 0.000 (0.000, 0.185), CFI = 1.000. However, the following paths were not significant (*p* > 0.05) and were therefore removed: direct paths from support seeking, social support, *n*Aff, and *n*Ach to VE, direct path from *n*Aff to social support, from *n*Ach to support seeking, and from *n*Ach to chronic stress. Furthermore, *n*Pow was completely removed from the model as none of its paths were significant.

**Figure 1:**
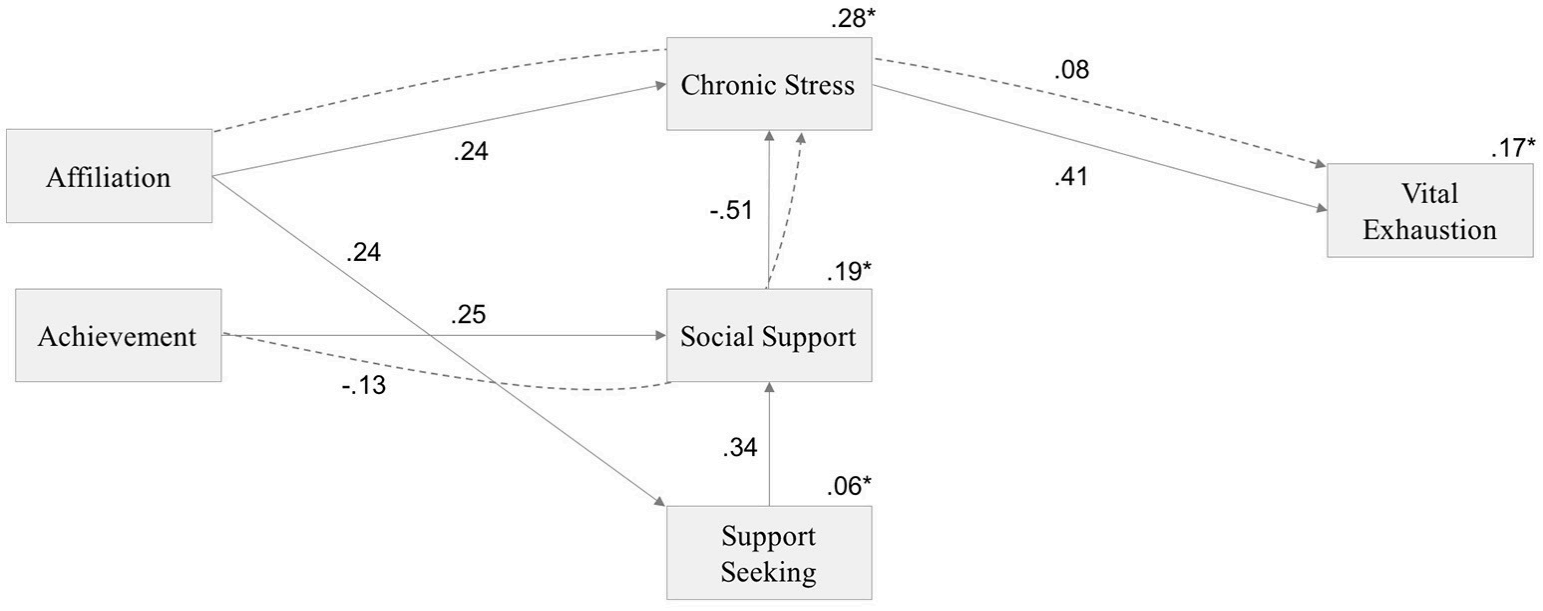
Structural equation model showing direct and indirect relationships between implicit motives, support seeking, perceived social support, perceived chronic stress, and vital exhaustion. Solid arrows indicate direct effects. Dashed arrows show indirect effects of achievement on chronic stress through the mediator social support as well as the indirect effect of affiliation on vital exhaustion through the mediator chronic stress. Significant standardized regression coefficients are shown in the numbers adjacent to the arrows. Numbers with asterick represent variance explained.

The final model as shown in Figure 1 showed a very good fit to the data: χ^2^(8, N = 101) = 4.896, *p* = 0.769, χ^2^/*df* = 0.612, RMSEA = 0.000 (0.000, 0.081), CFI = 1.000. Direct and indirect effects are reported in Table 3. The indirect effect of affiliation motive on VE through chronic stress was *b* = 0.079 (0.036, 0.019), *p* = 0.012. Furthermore, affiliation motive showed an indirect negative effect on VE through higher support seeking and higher social support and consequently lower chronic stress, *b* = −0.014 (−0.051, −0.003), *p* < 0.05.

**Table 3:** Direct and indirect effects and 95% confidence intervals for the overall structural equation model.

	***B***	***SE***	**95% CI**		***p***
			**Lower bound**	**Upper bound**	
**DIRECT EFFECTS**					
AFF → CS	0.236	0.079	0.073	0.384	0.005
AFF → SS	0.243	0.095	0.046	0.421	0.018
ACH → PSS	0.253	0.084	0.077	0.409	0.006
SS → PSS	0.342	0.093	0.152	0.512	0.001
PSS → CS	−0.505	0.085	−0.654	−0.317	0.000
CS → VE	0.407	0.090	0.211	0.568	0.000
**INDIRECT EFFECTS**					
AFF → CS → VE	0.079	0.036	0.019	0.162	0.012
AFF → SS → PSS	0.083	0.043	0.016	0.187	0.011
AFF → SS → PSS → CS	−0.041	0.024	−0.109	−0.008	*
AFF → SS → PSS → CS → VE	−0.014	0.011	−0.051	−0.003	*
ACH → PSS → CS	−0.128	0.047	−0.230	−0.043	0.004
ACH → PSS → CS → VE	−0.046	0.024	−0.115	−0.013	*

N = 101. AFF, Implicit affiliation motive; ACH, Implicit achievement motive; CS, Chronic stress; PSS, Perceived social support; SS, Support seeking; VE, Vital exhaustion. *Indirect effects with two or more mediators were tested in PROCESS. No significance levels are given. Instead, significant effects are indicated by bootstrapped confidence intervals not including 0.

The indirect effect of achievement motive on chronic stress through social support was *b* = −0.128 (−0.230, −0.043), *p* = 0.011. Implicit achievement motivation further indirectly influenced VE through higher social support and lower chronic stress, *b* = −0.046 (−0.115, −0.013), *p* < 0.05. The overall model accounted for 6% of the variance in support seeking, 19% of the variance in perceived social support, 28% of the variance in perceived chronic stress, and 17% of the variance in VE.

### Moderation analyses

Analyses from the first part of this study showed that implicit motives influence the level of perceived chronic stress, perceived social support, and VE. For the third research question, we tested whether implicit motives might also serve as moderators of the relationship between perceived chronic stress and VE. Implicit motives did not moderate the relationship between a general measure of chronic stress and VE (*p* > 0.05). However, as indicated in the manual of the Trier Inventory of Chronic Stress (Schulz et al., 2004), the different subscales of chronic stress should be considered in order to determine which specific type of stress contributes to strain. Furthermore, previous research on implicit motives suggests that they become effective in situations that are relevant for the specific motivational domain (Wirth et al., 2006; Wegner et al., 2014).

Therefore, we ran moderation analyses for subtypes of chronic stress. We tested both implicit affiliation and achievement motives as moderators, as they were found to be relevant in the first part of this study, as opposed to implicit power motivation. The correlation analyses reported in Table 1 revealed significant positive relationships between VE and the following subscales of perceived chronic stress: *work overload, work discontent, excessive demands at work, lack of social recognition, lack of social contact*, and *chronic worrying*. We assumed that *lack of social recognition* would only be relevant in combination with implicit power, and thus removed this variable from our analyses*. Chronic worrying* was also not examined, as this scale is of a more general nature and the source of the worries cannot be determined (Schulz et al., 2004). Accordingly, VE was separately regressed on *work overload, work discontent, excessive demands at work*, and *lack of social contact*. Only models in which the interaction term reached significance are reported below.

#### Affiliation motive and work overload

**Figure 2:**
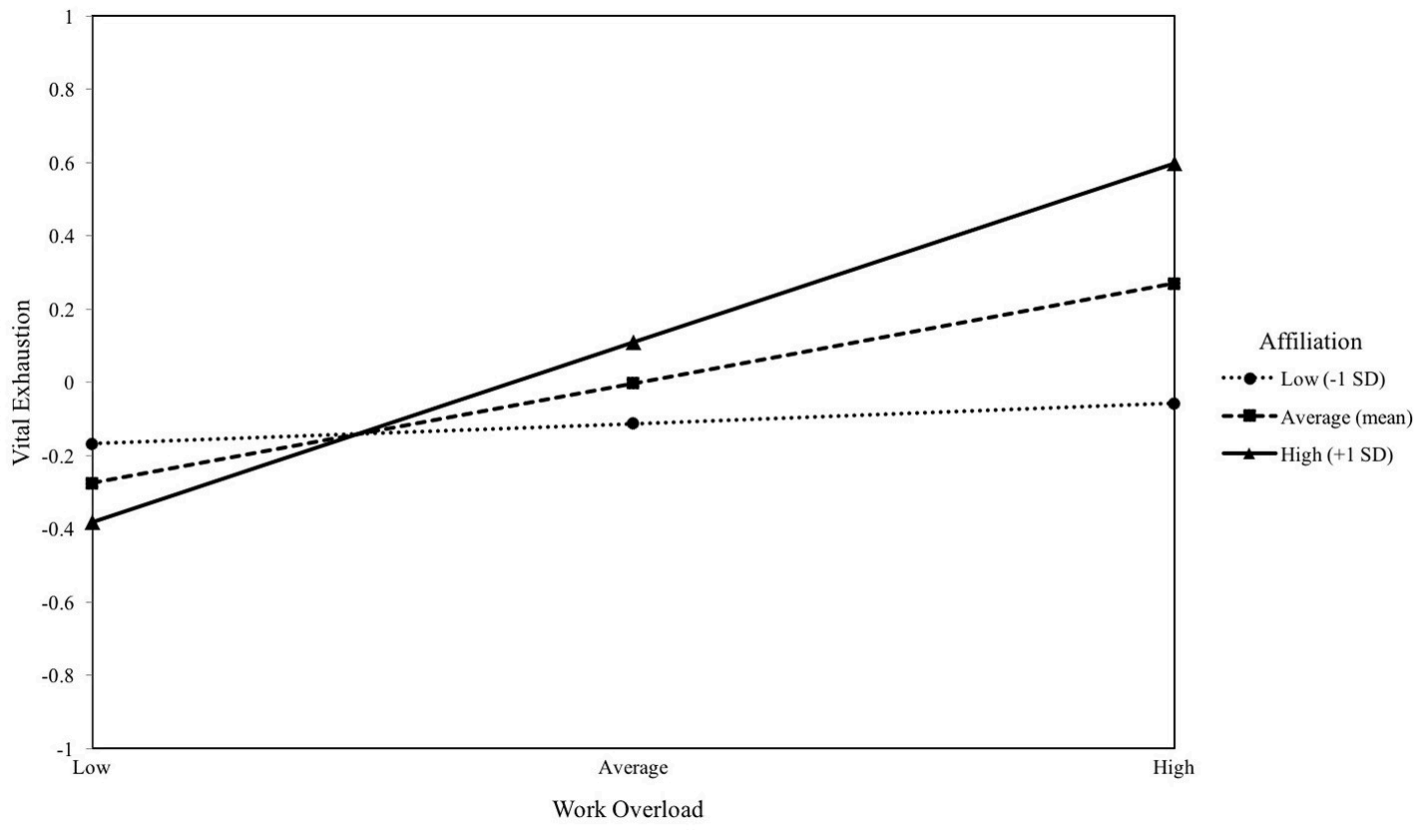
The moderating effect of implicit affiliation motive on the association between work overload and vital exhaustion. Simple slopes of work overload predicting vital exhaustion for 1 *SD* below the mean of affiliation, at the mean of affiliation, and 1 *SD* above the mean of affiliation.

Work overload was a significant predictor of VE (*b* = 0.27, *p* = 0.009). Implicit affiliation motive did not predict VE (*b* = 0.11, *p* > 0.05). However, the interaction between the independent variables was a significant predictor of VE (*b* = 0.21, *p* = 0.014). Inspection of conditional effects as shown in Figure 2 revealed that the effect of work overload on VE was only significant at average and high levels of affiliation motive (*b* = 0.27, *p* = 0.009 and *b* = 0.49, *p* < 0.001). The overall model was significant and explained 13% of the variance in VE [*F*_(6, 94)_ = 3.01, *p* = 0.009].

#### Affiliation motive and lack of social contact

**Figure 3:**
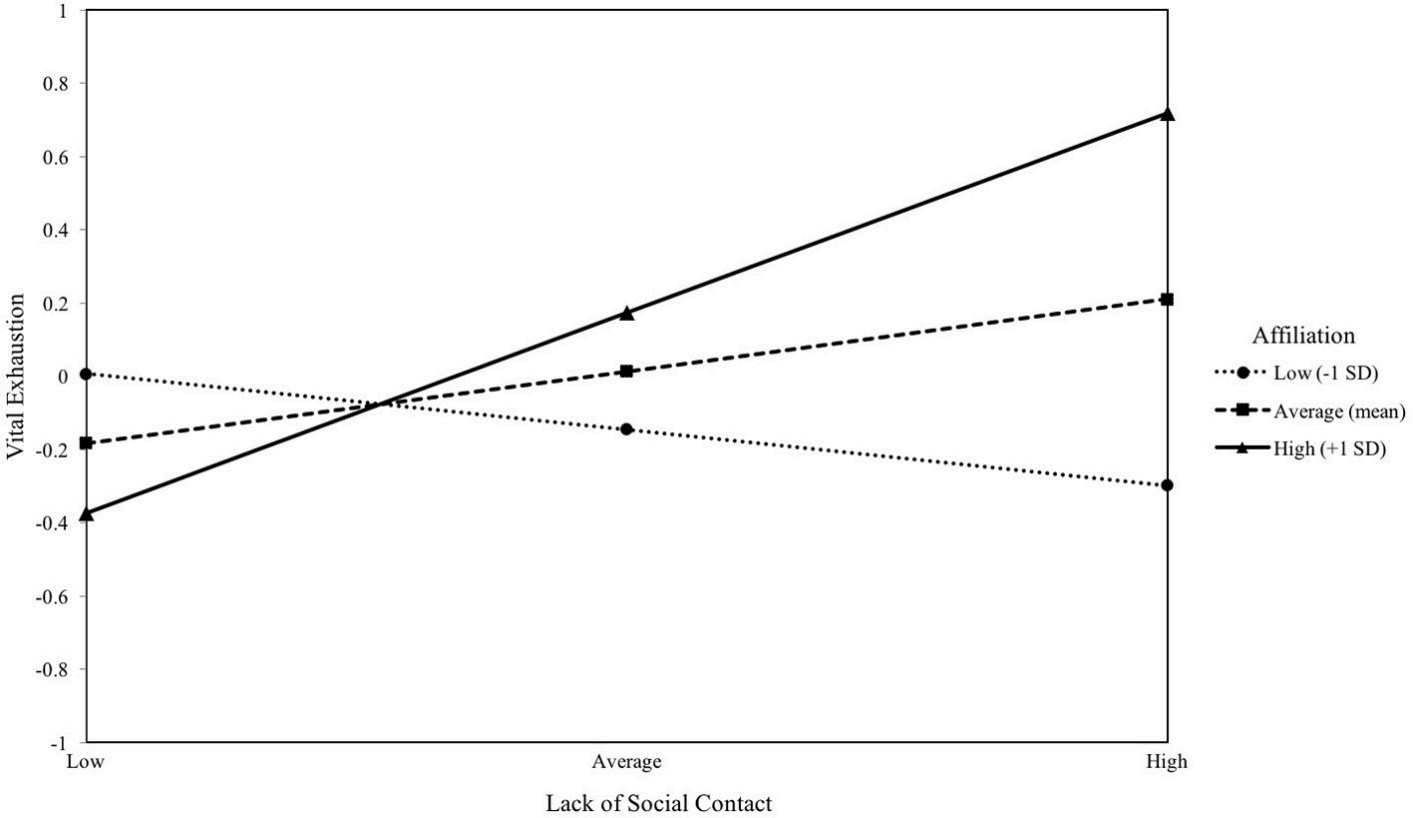
The moderating effect of implicit affiliation motive on the association between lack of social contact and vital exhaustion. Simple slopes of lack of social contact predicting vital exhaustion for 1 *SD* below the mean of affiliation, at the mean of affiliation, and 1 *SD* above the mean of affiliation.

Again, implicit affiliation motive was not a significant predictor of VE (*b* = 0.16, *p* > 0.05). Lack of social contact positively predicted VE (*b* = 0.20, *p* = 0.041). The interaction effect of affiliation motive and lack of social contact was a significant predictor of the dependent variable (*b* = 0.34, *p* = 0.002). This interaction is illustrated in Figure 3. Lack of social contact significantly predicted VE only in those individuals with an average affiliation motive (*b* = 0.20, *p* = 0.041) or one standard deviation above the mean (*b* = 0.55, *p* < 0.001). The model with all variables was significant [*R*^2^ = 0.15, *F*_(6, 94)_ = 4.15, *p* = 0.0010].

#### Achievement motive and work discontent

**Figure 4:**
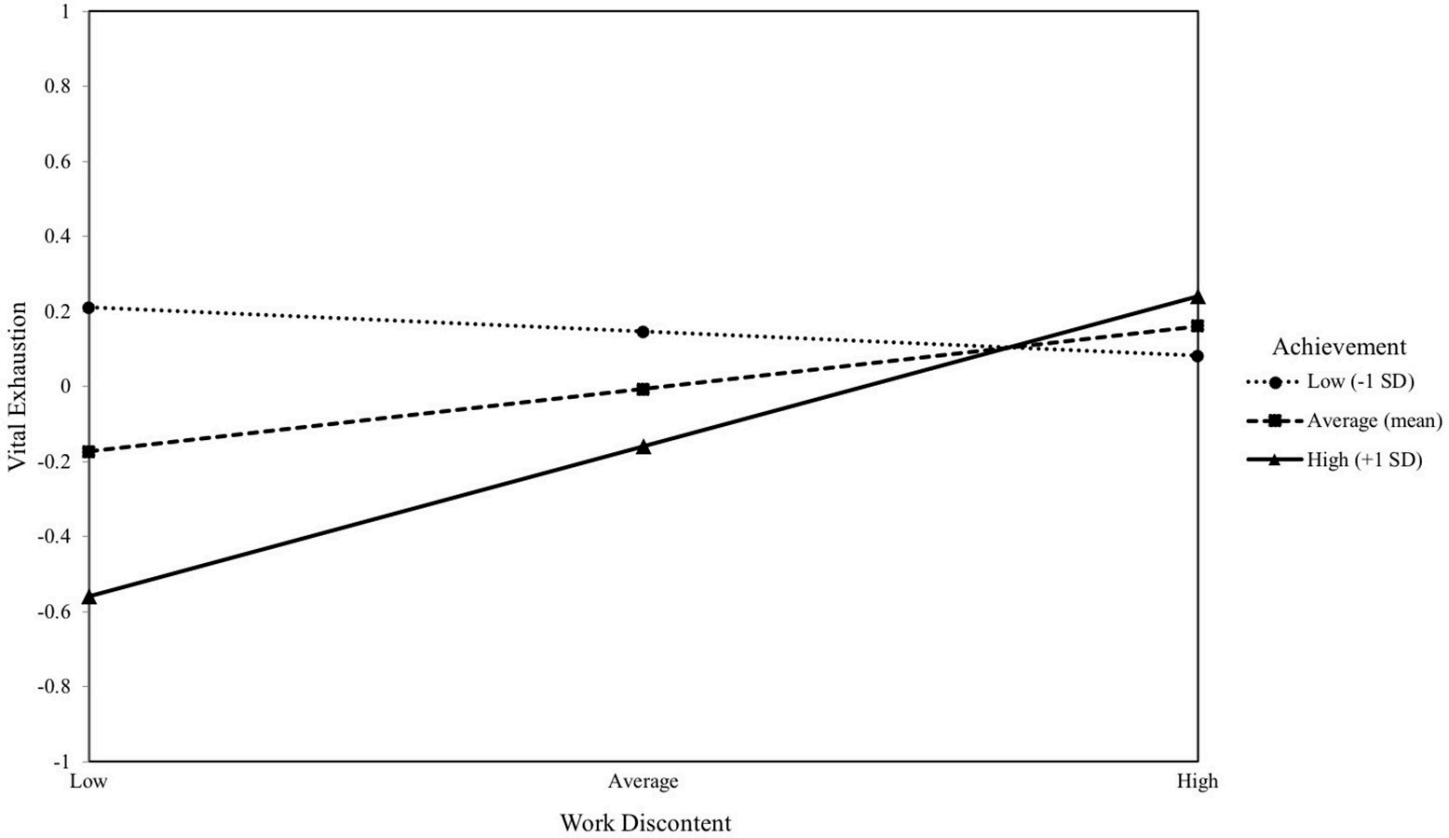
The moderating effect of implicit achievement motive on the association between work discontent and vital exhaustion. Simple slopes of work discontent predicting vital exhaustion for 1 *SD* below the mean of achievement, at the mean of achievement, and 1 *SD* above the mean of achievement.

VE was not predicted by implicit achievement motive (*b* = −0.15, *p* > 0.05) or by work discontent (*b* = 0.18, *p* > 0.05). In contrast, the interaction term between achievement motive and work discontent significantly predicted VE (*b* = 0.23, *p* = 0.019). Work discontent was a significant predictor of VE only at high levels of the moderator, as displayed in Figure 4 (*b* = 0.41, *p* = 0.002). However, the overall model including all control variables was not significant [*R*^2^ = 0.11, *F*_(6, 94)_ = 1.99, *p* = 0.074].

Removing the non-significant control variables resulted in the model reaching significance, with only minor changes in standard coefficients of the independent variables and the interaction term. This altered model explained the same amount of variance in VE [*R*^2^ = 0.11, *F*_(3, 97)_ = 4.02, *p* = 0.009].

#### Achievement motive and lack of social contact

**Figure 5:**
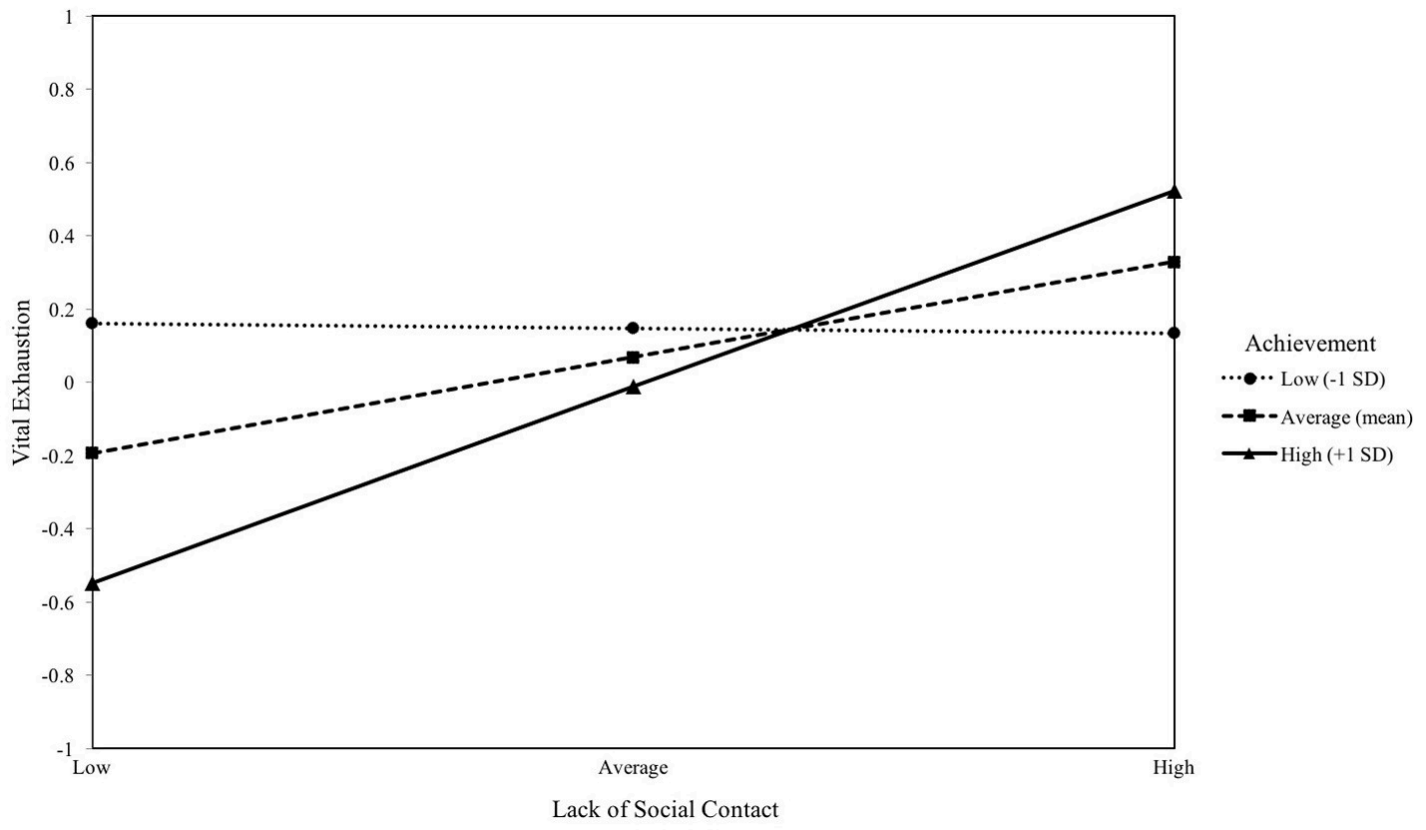
The moderating effect of implicit achievement motive on the association between lack of social contact and vital exhaustion. Simple slopes of lack of social contact predicting vital exhaustion for 1 *SD* below the mean of achievement, at the mean of achievement, and 1 *SD* above the mean of achievement.

As in the previous models, lack of social contact was a significant predictor of VE (*b* = 0.26, *p* = 0.018), unlike implicit achievement motive (*b* = −0.08, *p* > 0.05). The interaction effect significantly predicted VE and is illustrated in Figure 5 (*b* = 0.27, *p* = 0.042). The effect of lack of social contact on VE was significant at average and high levels of achievement motive (*b* = 0.26, *p* = 0.018 and *b* = 0.54, *p* < 0.001). The regression model explained 11% of the variance in VE [*F*_(6, 94)_ = 2.21, *p* = 0.049].

## Discussion

### Summary of results

This study addressed the role of implicit motives in the experience of chronic stress, social support, and VE in middle-aged men. In the first research question, the relationship between VE and specific types of chronic stress was analyzed. VE was positively associated with work-related stress, namely work overload, excessive demands at work, and work discontent. Furthermore, men who reported chronic worrying also experienced more symptoms of exhaustion. In terms of social stress, lack of social recognition and social contact were positively related to VE. In contrast, support-seeking behavior and perceived social support were linked to lower levels of VE.

Implicit motives were hypothesized to potentially exert a direct and indirect influence on the perception of chronic stress, social support, and the level of VE. The analyses for the second research question revealed that implicit affiliation motivation was positively associated with VE through increased perceived chronic stress. The effect of the affiliation motive on VE became negative when the pathways through support seeking and perceived social support were considered. Likewise, implicit achievement motivation was linked to lower VE through higher perceived support and less chronic stress. We found no direct correlations between implicit motives and VE or between implicit power and chronic stress or social support.

As proposed in the third research question, implicit motives further moderated the relationships between subtypes of stress and VE. Affiliation motivation increased the negative effect of work overload and lack of social contact on VE, while achievement motive enhanced the negative association between VE and lack of social contact and work discontent, respectively^1^.

### Interpretation of results

The present findings indicate that specific subtypes of perceived chronic stress are related to the experience of VE. Even though several of these stressors are rooted in the context of work, stressors inherent in the social environment have been shown to impair mental health as well.

Work overload and excessive demands at work were both positively correlated with VE. Similar results have consistently been reported for emotional exhaustion, a component of burnout (Alarcon, 2011; Qaiser et al., 2015). With respect to VE, only one other study showed a positive association between workload and VE in a sample of industrial employees (Schnorpfeil et al., 2002). The positive association between work discontent and VE represents a new finding. A meta-analysis previously showed that job satisfaction was negatively related to emotional exhaustion (Alarcon, 2011). According to the authors of the stress inventory used in this study, discontent arises when tasks need to be completed that do not meet one's own interest or for which one's abilities cannot be used (Schulz et al., 2004). Therefore, improving the fit between an individual's interests and skills and the characteristics and demands of their tasks should improve satisfaction with work. Moreover, work discontent was positively correlated with lack of social recognition and social contact. Thus, the social environment at least co-determines whether a person is satisfied with his/her work.

Humans rely heavily on social relationships and cues from their social environment. In our study, social support appeared to be a protective factor against the experience of stress, and consequently, VE. However, the present sample had slightly lower ratings of perceived support compared to those in other studies (Schulz and Schwarzer, 2003; Wirtz et al., 2006). Individuals with low support are at a higher risk of VE and heart failure (Cené et al., 2012). In this study, perceived lack of social contact was also positively associated with VE. The correlations further suggest that lack of social contact might arise from work overload and excessive demands at work. Being absorbed in work not only decreases the time spent with colleagues at work but also reduces the interactions with friends and family in one's private life. Furthermore, lack of social recognition was associated with higher VE. Previous research showed that lack of social recognition is responsible for the experience of stress and burnout (Lowenstein, 1991; McLean and Andrew, 2000). Recognition involves appreciation both of one's work and of one's person. Creating a work culture that shows appreciation for an individual's achievements on a regular basis, without losing sight of the person behind the success, might help to reduce the risk of feeling exhausted.

Finally, chronic worrying was strongly related to the degree of VE. Perseverative worry is involved in anxiety disorders and depression, but is also a common stress response in non-psychiatric populations (Tallis et al., 1992; Ladouceur et al., 1999). However, chronic worrying prolongs the physiological stress response, and is thus related to impairments in health and well-being (Brosschot and van der Doef, 2006; Brosschot et al., 2006). For example, worrying was found to be a significant predictor of coronary heart disease in men (Kubansky et al., 1997). Mindfulness-based interventions have shown to be effective in reducing chronic worrying and the degree of stress and burnout (Delgado et al., 2010; Roeser et al., 2013). In sum, chronic worrying appears to be an important aspect of VE. Nevertheless, longitudinal studies need to clarify whether worrying is a cause or a consequence of VE.

It is important to note that the present sample differs from other study populations with respect to the degree of VE and the perception of chronic stress. Compared to other male samples of a similar age range, on average, the participants in this study reported being more vitally exhausted (Schnorpfeil et al., 2002; Rafael et al., 2014). However, participation in this study was restricted to levels of vital exhaustion of 4 or higher compared to other studies allowing a range of 0–18. Interestingly, despite lower mean scores of VE in the present participants, their mean perception of chronic stress was lower than the mean of a representative German sample, with the exception of chronic worrying (Petrowski, 2012). Presumably, it is not the quantitative experience of stress *per se* that leads to signs of VE, but rather other processes involved, such as an individual's implicit motives.

The present results suggest that individuals high in implicit affiliation motivation are more prone to experience stress and VE. This is in contrast to a previous study that found a buffering effect of implicit affiliation motive on the perception of constraint due to fatherhood in a sample of fathers (Ruppen et al., 2016). These divergent findings are likely the result of the interaction between motives and the specific situation. Apparently, fatherhood offers incentives to satisfy affiliative needs (e.g., playing with your child). Therefore, these men do not perceive their paternal role as stressful. The present results suggest that men in this sample had difficulties to fulfill their affiliative needs or that they experienced threats to their implicit affiliation motivation. This motive frustration would be expressed in higher perceived chronic stress as seen in the present findings.

Moderation analyses further confirmed that adverse effects for an individual's health arise when a person motivated for affiliation is confronted with motive-relevant types of stress. When individuals have to deal with an excessive amount of work (*work overload*), they may have to work overtime and have less leisure time available for the maintenance of social relationships. This would be more detrimental for individuals high in affiliation motivation as they have less time and fewer opportunities to satisfy their affiliative needs through social contact, resulting in higher exhaustion.

Likewise, implicit affiliation motive increased the negative relationship between lack of social contact and VE. Individuals motivated for affiliation are vulnerable to separation and rejection, to which they react with negative emotions such as sadness (McClelland et al., 1989). Wirth and Schultheiss (2006) found that participants' cortisol levels increased after they had watched an affiliation-themed film which contained strong elements of rejection (Wirth and Schultheiss, 2006). Hence, lack of social contact is threatening to the need for affiliation, and thus impairs well-being.

However, the negative effect of affiliation motive on VE in the structural queation model diminished when social support was taken into account. Individuals higher in affiliation motivation apparently seek and perceive more social support. Upon experiencing stress, the steroid hormone progesterone is released, promoting affiliative behavior and in turn helping the individual to adjust to a stressor (Taylor, 2009; Wirth, 2011). Showing tend-and-befriend behavior in the face of stress is more characteristic of women than of men, although not exclusively so (Taylor, 2009). It is likely that men who are highly motivated for affiliation also favor this type of stress response.

Contrary to expectations, implicit achievement motivation was linked to more perceived support, and thus lower chronic stress and VE. A high need for achievement further increased the negative influence of lack of social contact on VE. These findings are rather surprising, since achievement motivation has rarely been considered in studies on social relationships. However, achievement motive is shaped in childhood in response to parents' reactions to the success or failure in mastering challenges (McClelland and Pilon, 1983). Therefore, social signals should remain important for achievement motivated individuals in adulthood (Stanton et al., 2010). Support for this assumption comes from a re-analysis of an experimental study by Schultheiss and Hale (2007): Participants with a higher need for achievement directed their attention more toward joyful faces as opposed to surprised or angry faces. Furthermore, certain tasks can only be accomplished by collaborating with others (Pang et al., 2009). Hence, lack of social contact might prevent a person from satisfying their need for achievement. Nevertheless, this line of argument remains speculative, and further studies are needed to determine the meaning of this finding.

Implicit achievement motive further exacerbated the negative relationship between work discontent and VE. This finding is in contrast to the study by Schultheiss et al. (2014), which showed that high achievement motivation attenuated the acute stress response in a competitive task. In general, individuals motivated for achievement view difficulties as challenging rather than stressful (Reeve et al., 1987). These results suggest that different mechanisms could be relevant when individuals motivated for achievement are exposed to chronic as opposed to acute stress. Alternatively, the influence of implicit achievement motivation could differ with regard to self-reported and biological measures of stress and health.

A number of studies have shown that implicit power motive is involved in an individual's health and well-being (Wirth et al., 2006; Hall et al., 2010; Ruppen et al., 2016). In the present sample, however, implicit power motive was unrelated to any other variables. Different explanations for this divergence can be considered. In general, the work environment should provide various incentives to satisfy power-related needs. Therefore, power-motivated men might not have experienced their work to be as stressful compared to individuals who are more strongly concerned with affiliation or achievement in the first place. Potentially, these men did not take part in the study as they did not feel addressed by the wording in the recruiting process. This would offer an explanation for the raw power motive scores which are lower than those reported in other studies (e.g., Pang and Schultheiss, 2005). Moreover, in men, their power motive usually is stronger than their motive for achievement or affiliation. This is not the case for the present data which also reveal an unusually high affiliation motivation. Therefore, the lack of power effects could be due to specific characteristics of the sample. Most participating men in this study were well-educated, employed full-time, had an above-average income, and were in a stable relationship. Alternatively, the influence of activity inhibition should be taken into account. Adverse health effects have often been reported for inhibited power motivation, that is a combination of high implicit power motive and high activity inhibition (Schultheiss et al., 2009). However, *post-hoc* analyses with the present data revealed no significant moderating effect of activity inhibition on the relationship between implicit power motivation and subtypes of chronic stress or VE.

### Limitations and future research

A major strength of this study is the combination of self-reported data and projective measures. Furthermore, we assessed a range of subtypes of perceived chronic stress, which permitted a more detailed analysis of the stressor-strain relationship. Yet, our results are subject to several limitations. The cross-sectional design of the study does not allow for conclusions about the direction of causality of the relationship between chronic stress and VE. Possibly, men who were more vitally exhausted in the first place also experience more chronic stress due to their exhaustion. However, the causal directions assumed in the present study are based on a theoretical framework and have been reported in longitudinal research as well (Godin et al., 2005; Chandola et al., 2006; Rod et al., 2009). Nevertheless, a replication of our results in studies using a longitudinal design would strengthen the value of our findings. Certain data in this study showed non-normal distributions which did not result from any obvious data errors. To address this problem, non-parametric analysis procedures were used throughout the study. Overall, a larger sample size would contribute to normality as well as stability of our effects. The results in this study can be considered small to medium in effect (Cohen, 1990; Warner, 2013). As (Schönbrodt and Perugini, 2013) have shown in their systematic analysis, correlations become stable at samples consisting of 250 cases or more. That is, in samples with fewer participants a statement can be made regarding the direction but not the size of an effect. Moreover, low statistical power may increase the risk of both type I and type II error (Christley, 2010; Benjamin et al., 2018). Therefore, larger-scale studies should be conducted to answer questions regarding stability and actual strength of the relationships analyzed in this paper.

Furthermore, the reported findings only apply to employed men aged 40–65 years who showed signs of VE. Other populations might differ with regard to the experience of stress, support, VE, and the role of implicit motives in these processes. With regard to gender, women generally report higher VE than men, which could be due to biological and psychological sex differences in stress response (Verma et al., 2011; Frestad and Prescott, 2017). The male stress response is generally characterized as a fight-or-flight reaction, while women more often show tend-and-befriend behavior (Taylor et al., 2000). The latter buffers the physiological stress response. Accordingly, in women, an attenuated activation of the sympathoadrenal system and responsiveness of the hypothalamic-pituitary-adrenal axis has been observed, resulting in a delayed and thus, less effective stress response (Verma et al., 2011). Moreover, women show more maladaptive cognitive and coping styles than men which pose an additional risk for the development of mental disorders (Butler and Nolen-Hoeksema, 1994; Mazure and Maciejewski, 2003; Matud, 2004). Gender differences have also been described for the size of social networks, the perception and mobilization of social support, as well as its beneficial effects (Taylor, 2009). Particularly in times of stress, women fall back on their sources of social support much more than men do. Gender effects are observed in implicit motives as well. A recent review concluded that women generally have a higher implicit affiliation motive but do not differ from men with regard to implicit achievement or power motivation (Drescher and Schultheiss, 2016). Moreover, sex hormones or menstrual cycle phase influence the expression of implicit motives (Schultheiss et al., 2003; Ball et al., 2014).

As outlined above, mean VE scores were higher compared to results reported in previous studies. In contrast to other studies on VE, participation in the present study was restricted to individuals with a VE score of 4 or higher (e.g., Schnorpfeil et al., 2002). Arguably, other results might have emerged in a sample that included individuals without or very low levels of exhaustion. This could be particularly true with respect to the missing effects of implicit power motivation. However, the larger project specifically set out to investigate vital exhaustion in middle-aged and senior men and recruiting was designed accordingly. From a total of 245 men that did not pass the screening prior to study participation, about half decided to drop out themselves (*n* = 116). A similar number of men had to be excluded due to inclusion or exclusion criteria (*n* = 119). In the end, only 10 men were excluded because they did not pass the cut-off score of VE. With these differences in gender and exhaustion state in mind, the findings presented here cannot be generalized without future studies extending their investigation to other populations as well.

## Conclusion

This study adds to the growing literature on VE by identifying specific types of perceived chronic stress and their individual association with signs of exhaustion. Besides work stress, the social environment is an important factor to consider, as it constitutes both a resource and a source of stress. The present findings offer starting points for stress intervention studies. In particular, the focus should be on providing opportunities for social exchange and improving social relationships.

Furthermore, first evidence was provided for the role of motivational aspects of personality in the stress-strain relationship. The implicit motives for affiliation and achievement play a key role with respect to the experience of chronic stress, social support, and VE. Consequently, an individual's implicit motives need to be considered when planning prevention or intervention measures. In conclusion, this study contributes to a better understanding of the individual differences in the experience of stress and support, and the development of stress-related psychological health outcomes.

## Data availability

The data of this study cannot be shared due to ethical reasons. The participating men were not informed of this possibility and accordingly did not agree to share their data publicly when they were providing informed study participation consent.

## Ethics statement

The study Men Stress 40+—Buffering the effects of vital exhaustion in men 40+ was approved by the Cantonal Ethical Committee of Zurich on the 2nd November 2015 (KEK-ZH-Nr. 2015-0446).

## Author contributions

JS, EN, and UE were involved in the study design and planning. JS coded the text samples for implicit motives and was responsible for data analysis and the draft of the manuscript. All authors reviewed, revised, and provided approval of the final version of the manuscript.

### Conflict of interest statement

The authors declare that the research was conducted in the absence of any commercial or financial relationships that could be construed as a potential conflict of interest.

## Footnotes

^1^As suggested by a reviewer, moderation analyses were also computed without the inclusion of control variables age, education, and income since they did not significantly correlate with measures of stress or VE. Results did not change except for the moderation effect of implicit affiliation motivation on the relationship between lack of social contact and VE. Only high levels of affiliation instead of average and high levels did exacerbate the effect of lack of social contact on VE.
